# Spatial and temporal trends in the fate of silver nanoparticles in a whole-lake addition study

**DOI:** 10.1371/journal.pone.0201412

**Published:** 2018-08-15

**Authors:** Daniel C. Rearick, Lena Telgmann, Holger Hintelmann, Paul C. Frost, Marguerite A. Xenopoulos

**Affiliations:** 1 Environmental and Life Sciences Graduate Program, Trent University, Peterborough, Ontario, Canada; 2 Water Quality Centre, Department of Chemistry, Trent University, Peterborough, Ontario, Canada; 3 Department of Biology, Trent University, Peterborough, Ontario, Canada; VIT University, INDIA

## Abstract

Studies of the fate and toxicity of nanoparticles, including nanosilver (AgNPs), have been primarily conducted using bench scale studies over relatively short periods of time. To better understand the fate of AgNPs in natural aquatic ecosystems over longer time scales and ecological settings, we released suspensions of AgNPs (30–50 nm, capped with polyvinylpyrrolidone) into a boreal lake at the Experimental Lakes Area in Canada. Approximately 9 kg of silver was added from a shoreline point source from June to October 2014, which resulted in total Ag (TAg) concentrations of about 10 μg L^-1^ or less. In addition, dissolved Ag concentrations (DAg) were typically very low. Using single particle inductively coupled plasma mass spectrometry (sp-ICP-MS) analysis of grab samples, we found that the nanoparticles typically ranged in the 40–60 nm size class and were widely distributed throughout the lake, while larger aggregates (i.e. >100 nm) were infrequently detected. The highest occurrence of aggregates was found near the addition site; however, size distributions did not vary significantly among spatial locations or time suggesting rapid dispersal upon entry into the lake. Lake stratification at the thermocline was not a barrier to mobility of the AgNPs, as the particles were also detected in the hypolimnion. Environmental factors influenced Ag size distributions over sampling locations and time. Total dissolved phosphorus, bacterioplankton chlorophyll-a, and sampling time strongly correlated with aggregation and dissolution dynamics. AgNPs thus appear to be relatively mobile and persistent over the growing season in lake ecosystems.

## Introduction

Silver nanoparticles (AgNPs) are used in numerous products including additives to textiles, medical equipment, antibacterial creams and consumer goods [1]. The potential release of AgNPs into rivers and lakes is predicted to be in the ng L^-1^ range [2], although more recent modelling estimates show concentrations up to 70 times higher are possible due to the rapid increase in production volumes [3]. There are few data on measured environmental concentrations of AgNPs that can be compared to these predicted estimates [4]. Our knowledge on the fate and effects of AgNPs in aquatic ecosystems is thus limited with much of information derived from short-term experiments completed in an artificial settings [5]. While recent efforts have advanced our understanding of factors that drive AgNP persistence, transformation, and toxicity; it is evident there is a need to add greater realism to research examining environmental fate of AgNPs [6].

Nanoparticle transformations in surface waters are driven by chemical, physical, and biological processes affecting particle transformation. Such transformations can lead to particles remaining in nanoparticle form, dissolution to Ag^+^, adsorption to macromolecules or cells and agglomeration or aggregation. Particle form in turn affects movement in the aquatic environment as well as toxicity and bioavailability [7–9]. A number of chemical and physical conditions affect transformation of AgNPs including ionic strength [10–12], the availability of ligands such as dissolved organic carbon [13,14], sulfide [6], chloride [15], and phosphate [16], as well as light [17], oxidation conditions [18] and pH [11,14]. In a natural environmental setting, such factors may influence AgNPs in a complex nonlinear way through the interplay of chemistry, physics and biology.

Our knowledge of the fate and effects of AgNPs in aquatic ecosystems has been primarily generated from bench scale studies conducted over the short term in the absence of interacting environmental variables [5]. Relatively few studies have examined AgNP responses to interactive environmental conditions under more natural settings. These studies include in short-term experiments (i.e. <1 week) conducted in microcosms [9,19] and in larger mesocosms [20]. Over longer time scales ranging up to several weeks, AgNP fate has been studied in a wetland mesocosm [8], in mesocosms placed in a lake [21–24] and in an estuary [25]. In general, these studies in mesocosms and microcosms indicate that the concentrations of Ag^+^ are low in the natural aquatic environment because of rapid binding to organic and inorganic ligands [20,21,25]. Between these studies though the persistence of AgNPs in the water column appears to vary substantially. Within wetland mesocosms, the concentrations of AgNPs in the aqueous phase declined by approximately 20% after 24 hours [20]. Removal from the water column was even faster in estuarine mesocosms where all detectable AgNPs were gone within 6 to 12 hours [25]. This estuary result is easily explained by the poor stability of nanoparticles in water under conditions of high ionic strength. However, in mesocosms deployed in a lake with low ionic strength and high levels of dissolved organic carbon, AgNPs added to the water column were relatively persistent, with a half-life of approximately 20 days [21].

While these data have improved our understanding of AgNP persistence, transformation, and toxicity, we need more studies under environmentally realistic scenarios to better define the fate of AgNPs in aquatic ecosystems [6]. Nanoparticle fate and transformation has yet to be assessed at ecosystem scales where physical mechanisms affecting particle dispersal and transformation, such as currents and thermal stratification may be important determinants of nanoparticle fate. A vertical temperature gradient may reduce the transport of AgNPs to the hypolimnion during summer stratification and result in a greater proportion of cell-bound or larger aggregates settling to this region. It has been shown that vertical movement of dissolved materials during thermal stratification is low with movement limited to settling particles and precipitates [26]. However, spring and fall mixing events would likely redistribute Ag species that were separated by thermal stratification, and in turn alter organismal exposure to various AgNP forms seasonally. AgNP transport could also be strongly affected by wave action and underlying currents driven by local weather conditions.

In the present study, we describe the distribution of AgNPs in a whole lake ecosystem in which AgNPs were added via a point source over four months in 2014. Our main objective was to investigate the size distributions of AgNPs over spatial and temporal scales in an entire lake using single-particle inductively coupled plasma mass spectrometry (sp-ICP-MS) [27]. This is an effective analytical technique to quantify AgNP size distributions at low concentrations (μg L^-1^ and ng L^-1^) in aquatic matrixes [28,29]. Secondly, we sought to quantify changes within AgNP size classes as well as total dissolved Ag across spatial locations and through time using synchrony analysis. Lastly, we used partial least squares regression analysis to compare Ag size classes to environmental parameters in order to understand the importance of each factor toward explaining the observed particle size distributions. Our findings provide insight to AgNP fate and structure in freshwater ecosystems.

## Materials and methods

### Experimental design

Research using the IISD-ELA was made possible by special federal (Canada) and provincial (Ontario) legislations (Minister of Justice, Experimental Lakes Area Research Activities Regulations, SOR/2014-95 and Environmental Protection Act, R.S.O. 1990, c. E.19, O. Reg. 60/14: Experimental Lakes Area). Permission to conduct the whole lake experiment was granted by IISD-ELA on May 21, 2014. Permission to access the field station through a closed road (Pine Road) was granted through a Travel Permit from the Ontario Ministry of Natural Resources.

AgNP suspensions were added to Lake 222 at the Experimental Lakes Area (ELA) located in northwestern Ontario, Canada (49°41'42.0"N; 93°43'27.7"W) from June to late August in 2014. ELA is an isolated region with pristine lakes devoid of human influence that are used for long term monitoring and whole ecosystem experiments. Lake 222 is a dimictic, oligotrophic lake present on the Canadian Shield with a maximum depth of 5.8 m. The surface area is estimated to be approximately 180,000 m^2^ with a volume of 6 x 10^5^ m^3^. The lake stratifies during the summer to form an upper epilimnion and a lower hypolimnion with the top of the thermocline forming at about a 2 m depth. During the experiment, dissolved organic carbon (DOC) within the lake ranged from 9 to 16 mg L^-1^ with circumneutral pH and conductivity ranging from 15–30 μS cm^-1^.

### AgNPs preparation and addition

AgNPs capped with polyvinylpyrrolidone (PVP) were used for the experiment and all suspensions were prepared onsite at ELA. Spherical AgNPs (99.9% purity, 5–10 m^2^ g^-1^ specific surface area, 0.2% by weight PVP surface coating) with a hydrodynamic diameter of 30–50 nm, as reported by the manufacturer were purchased in powder form from NanoAmor (TX, USA). These nanoparticles were suspended in filtered lake water (0.8 μm) with 0.025% gum arabic added as an additional stabilizer. Suspensions were created using a rotor-stator dispersion mill (Model L) purchased from Kady® International (Scarborough, ME, USA) at a final concentration of 5.2 g L^-1^ AgNPs. The methods used to suspend the AgNPs and the size characteristics of the particles in suspension are described in detail elsewhere [30]. Analysis by dynamic light scattering indicated that the nanoparticles were stable in suspension for 2 weeks after mixing, and were primarily in the 30–50 nm size range, although there was evidence of agglomerates present with sizes in the 200 nm range [30]. The suspension was stored in the dark overnight at 4°C before transportation to the lake for addition via a pump anchored on the southwestern shoreline. The additions began on June 15, 2014 and continued until October 20, 2014, for a total addition of approximately 9 kg of silver.

### Ag sample collection

Grab samples of lake water were collected at weekly intervals at 6 locations around the lake from June to mid-August, with an additional sampling event after the fall turnover in October. Samples were collected via a Van Dorn bottle and screened through a 35 μm mesh to remove coarse organic debris and zooplankton. Sites selected to assess the horizontal distribution included near the point source, 0.1 km from point source, center buoy surface, and lake outflow. Samples to assess the vertical distribution of AgNP were collected at the center buoy location at the deepest point of the lake in the epilimnion (surface water), the metalimnion (at the thermocline of 2–2.5 m depth), and the hypolimnion (at 4–4.5 m depth). Upon collection, all samples were flash frozen with liquid nitrogen and stored at -80°C until analysis. Our previous studies showed that flash freezing was an effective method for preserving the size distribution of AgNPs in aqueous suspensions [21].

### Materials

Aqueous Ag(I) standard for ICP-MS (1000 mg L^-1^) analysis was purchased from SPC Science (Baie D’Urfé, QC, Canada). A suspension of PVP-coated AgNPs with a spherical shape and a diameter of 80 nm was purchased as a reference standard from nanoComposix (San Diego, CA, USA). Nitric acid (65%) was purchased from BDH Chemicals through VWR International (Radnor, PA, USA). All chemicals were used in the highest quality available. Water was purified with a Milli-Q Element system (Millipore, Billerica, MA, USA). A working suspension of AgNPs with a concentration of 20 μg L^-1^ was prepared daily by dilution of the nanoComposix stock suspension with water. The Ag ICP-MS standard solution was diluted to a 100 μg L^-1^ working solution.

### Limnological variable collection and assessment

During each sampling event, temperature (°C) and dissolved oxygen (mg L^-1^) were measured at 0.5 m vertical increments to establish the stratification depth at the center buoy site (maximum depth location) using a calibrated YSI 550A Dissolved Oxygen Instrument (Yellow Springs, OH). Buoyancy frequency was calculated using the “rLakeAnalyzer” package in R by incorporating lake depth and temperature data from each date. Light profiles were taken at the surface and at 0.5 m incremental depths using a Licor LI 1400 data logger (Lincoln, NE) to calculate light attenuation coefficients (Kd). Kd was calculated using the equation ln (E_2_/E_1_)/ (z_1_ -z_2_), where E_1_ was the surface irradiance (μmol s^-1^ m^-2^), E2 was the irradiance at the submerged depth, z_1_ was the surface depth (m) and z_2_ was the submerged depth (m). Water sample pH was measured using a calibrated (4 and 7 standards) Accumet Basic 15 pH meter (Fisher Scientific, Ottawa, Canada) in the lab.

Water samples for nutrient and carbon analyses were collected using a Van Dorn bottle and immediately screened (35μm) to remove large debris before being stored into a Nalgene bottle then placed in the dark and in a cooler before additional processing in the lab. Once in the lab, water was vacuum filtered through a 0.2 μm polycarbonate filter (Millipore, Toronto, Canada) to obtain water for TDN, TDP, (stored at -20°C) and DOC (ashed amber bottles stored at 4°C) samples until analysis. TDP (μg L^-1^) samples were determined by measuring P concentrations following persulfate digestions using a molybdate-blue colorimetric method [31] and a spectrophotometer (Cary-50, Varian, Palo Alto, California, U.S.A.). TDN (mg L^-1^) concentrations were assessed using a second derivative spectroscopy method [32] then measured on the Cary-50 spectrophotometer. DOC concentration (mg L^-1^) was measured following sample acidification on an OI Aurora TOC analyzer (Xylem, College Station, Texas, U.S.A) [33].

DOC absorbance was measured using a Cary-50 Varian Spectrophotometer (Palo Alto, CA). A blank of MilliQ was used as a reference for absorbance measurements. Specific ultraviolet absorbance (SUVA) was calculated using the measured absorbance at 254 nm and 280 nm (m^-1^) and dividing by the DOC concentration (mg L^-1^).

Chlorophyll-a was extracted using a cold ethanol technique following the EPA method 446.0 [34]. Briefly, samples were extracted using 95% ethanol for 24 hours in the dark. Excitation (440 nm) and emission (660nm) wavelengths were measured on a Varian Cary eclipse fluorescence spectrophotometer (Agilent Technologies, CA, USA). Spinach chl-a extract (Sigma Aldrich, MO, USA) was used to establish a standard curve. BP chla was obtained from pre-filtered water using a 1.2μm polycarbonate filter (Millipore, Toronto, Canada) by vacuum filtration using a GFF (0.7μm) filter (Whatman, NJ USA). Ses chla was obtained from pre-screened water (35 μm) through vacuum filtration using a GFF (0.7μm) filter (Whatman, NJ USA).

Bacteria abundance was determined using a SYBR Green Stain I (Invitrogen) with flow cytometry (Cytonomics FC 500 flow cytometer, Beckham Coulter, CA, USA) [35]. Samples were thawed and mixed, with 0.5 mL of the sample added to 10 μL SYBR green (10^−5^ dilution of stock) and 30 mM of potassium citrate followed by a 30-minute dark incubation before analysis. Bacterial cell counts were determined by correcting the flow cytometry output for reagent dilution and instrument flow rate and time analyzed. Final abundance was presented as bacterial cells x10^9^ L^-1^.

### Analysis of silver species

Prior to analysis, frozen samples were thawed in a water bath at room temperature. Measurements were started directly after thawing. Analysis of particles by sp-ICP-MS analysis, as well as analysis of total Ag was carried out with a ThermoFisher (Bremen, Germany) X-Series 2 ICP-MS. The samples were introduced into the plasma with a borosilicate glass conical nebulizer (1 mL/min, AHF, Tübingen, Germany). Aspiration was used to ensure the most stable signal during sp-ICP-MS analysis.

For total Ag analysis, sample introduction by peristaltic pump was chosen and the quadrupole ICP-MS was operated in peak hopping scan mode with a dwell time of 25 ms for monitoring of ^107^Ag, with data acquired in steady-state mode and processed using the Thermo PlasmaLab (Thermo Scientific) software. The operating parameters were manually optimized using 5 μg L^-1^ tuning solution containing the Ag(I) ICP-MS standard. The plasma was run at a RF power of 1450 W. Cool gas was operated with a flow of 15 L min^-1^. The spray chamber was cooled externally to 4°C. The run time for sp-ICP-MS analysis was 300 s for AgNP suspensions and dissolved Ag(I) solutions. External calibration using standard solutions from 0.1 to 200 μg L^-1^ of Ag spiked with In as an internal standard was used to generate a calibration curve. Standard solutions were prepared daily from a Ag stock by serial dilution in 4% nitric acid. The method detection limit for Ag was 0.07 μg L^-1^.

The sp-ICP-MS analysis was conducted essentially as described previously [28,36] in scan mode using a dwell time of 5 ms. A suspension of 80 nm PVP capped AgNPs with a defined concentration (i.e. 200 ng L^-1^) was prepared in water. The optimal flow rate of the nebulizer was determined daily. Data from the ICP-MS were processed using PlasmaLab software, version 2.5.9.300 (ThermoFisher). Raw data were exported as Thermo Electron Glitter Format V1.1. The numerical results were subsequently imported into the software OriginLab8 (OriginLab Corp., Northampton, MA, USA) for further processing. For transport efficiency determination, ^107^Ag intensities were plotted as histograms with increments of 1000 cps. The histograms were then fitted to a Gaussian function to determine the mean intensity of the nanoparticles in the samples. The mean intensities of the Ag(I) solutions used for calibration with Ag ICP-MS standard were determined by averaging the single readings from each analysis. The transport efficiency and mass flux calibration curve were calculated following established techniques [36]. The mean background intensity of all samples was determined by plotting ^107^Ag intensities with an increment of 200 cps. Individual NP sizes were calculated from their peak intensity by comparison to the mass flux calibration curve. The lower limit of particle sizes detectable under the operating conditions with this instrumentation was 45±5 nm.

Particles were then plotted by estimated diameter (nm) with number of events detected (S1 Fig). The results were then binned by particle sizes to include 40–60, 60–100, and >100 nm for further assessment. We selected these nominal size classes to represent particles remaining similar to initial AgNP form (40–60 nm), intermediate agglomerates ranging upward to approximately double the initial size (60–100 nm), and lastly larger aggregates defined as particles >100 nm.

During sp-ICP-MS analysis, dissolved silver is detected as a constant signal and can be distinguished from nanoparticle peaks [28]. Concentrations of dissolved silver (DAg) were calculated by analyzing the background ^107^Ag intensities obtained during AgNP determination using sp-ICP-MS. Recorded average mean background intensities were compared to signal intensities obtained from measuring Ag(I) standards. DAg concentrations were finally calculated from the calibration curve [36]. Note, that DAg in this study is therefore operationally defined as the dissolved Ag fraction that is not associated with AgNPs.

### Statistical analysis

Synchrony analysis was used to compare spatial and temporal coherence among silver patterns across sampling locations. Synchrony (S) was calculated among all spatial locations. Prior to analysis, data were z-transformed before estimation by averaging Pearson’s correlation coefficients from all pairwise comparisons among sites. Statistical significance was determined using a permutation test in R (package “synchrony”) with the observed mean S compared to a distribution of r values derived from n = 999 randomizations of the original time series data [37].

In addition to spatial-temporal analysis, we used partial least-squares regression (PLS) to model environmental variables (X) to estimate Ag size distributions (Y). The PLS approach is useful to characterize environmental factors by approximating X and Y variables to generate PLS components used to model the predictive relationship [38]. To present the predictive influence of X components on Ag size classes, we used variable importance in projection scores (VIP) [39]. VIP scores estimate the predictive contribution of each X-variable in the model and are weighted by Y-variance explained by each component. To interpret the relative contribution of VIP scores, we established criteria defining highly influential (VIP>1) and moderately influential (VIP = 1 to 0.8) based on previous approaches [40]. We presented the models using the variation explained by the first 2 PLS factors as weights to illustrate relatedness. Prior to model construction, all variables were tested for normality using Shapiro-Wilk’s test, and variables that did not meet statistical assumptions were log-transformed in SAS. Environmental variables were abbreviated as Julian day (Jday), dissolved oxygen (DO), temperature (Temp), DOC (dissolved organic carbon), total dissolved nitrogen (TDN), total dissolved phosphorus (TDP), bacterial abundance (Abund), bacterioplankton chlorophyll-a (BP chla), seston chlorophyll-a (Ses chla), Kd (light attenuation coefficient), buoyancy frequency (BV), abs at 280 nm (a280) abs at 254 nm (a254), Specific ultraviolet absorbance at 254 nm (SUVA), Specific ultraviolet absorbance at 280nm (e280) dissolved Ag (DAg), total Ag (TAg), and pH.

## Results and discussion

### Particle size distribution

**Fig 1:**
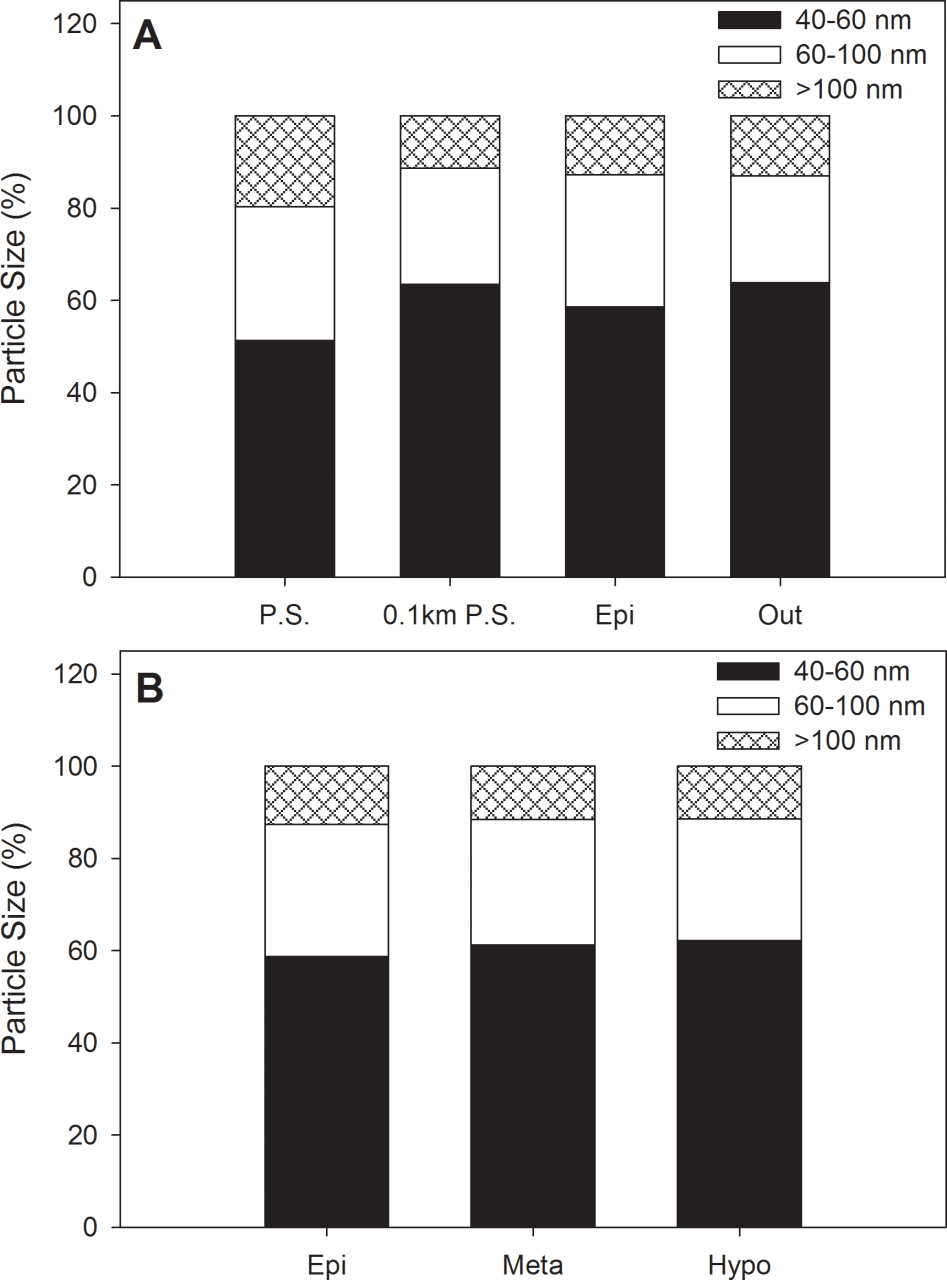
Mean annual particle size fractions across sampling site. Size distributions are presented as percent of total particles detected by averaging temporal intervals. Sites are presented as a surface horizontal gradient from the addition site to the lake outflow (A) and vertically within in the lake at the center buoy location (B). Site abbreviations are the following: Point Source (P.S)., 0.1km from the point source (0.1 km P.S.), littoral site near lake outflow (Out), epilimnion (Epi), metalimnion (Meta), and Hypolimnion (Hypo).

The size of AgNPs detected by sp-ICP-MS ranged from a lower size limit of ~40 nm up to particles greater than 100 nm. AgNPs were divided into ranges of 40–60 nm, 60–100 nm and >100 nm size classes. Over the period of AgNP addition to the lake from June 15 to October 20, 2014, the contribution of the 40–60 nm AgNP size class varied from 30 to 83% of the total particles detected. On average, this size class of particles comprised approximately 60% of the particles detected during the study (Fig 1), regardless of sampling location and time. This lack of change in AgNP form from the initial sized particles added into the lake can likely be attributed to the relatively high concentrations of DOC (11.39 ±1.64 mg L^-1^) and the AgNP capping agent. The stabilizing effect of DOM originates from these organic particles forming complexes with the nanoparticle surface, which alters the surface chemistry and reduces aggregation [13]. With the aqueous DOC (mg L^-1^) far exceeding the AgNP concentrations (low μg L^-1^) such reactions are likely to occur in this lake ecosystem [41]. Previous studies have demonstrated the stabilizing effects of DOC in microcosms [19] and mesocosms [20,21], with stabilizing effects detected in the presence of as little as 4 mg L^-1^ [13]. Additionally, the PVP capping agent can minimize aggregation kinetics compared to electrostatically capped and uncapped counterparts [11,42].

The intermediate size of 60–100 nm generally represented the second highest proportion ranging from 8–41%, while particles larger than 100 nm typically varied between 3 and 36% of particles examined (Fig 1). The scarcity of large aggregates across the lake may again be attributed to AgNP stability in the presence of organic matter [6,43]. Additionally, large organic particles binding to and forming AgNP aggregates may have favored deposition and rapid removal from the water column once formed [44]. It is interesting to note that the particle sizes in the 200 nm range that were present in the original suspension added to the lake [30] were not observed in the water column. The particles probably settled out of suspension relatively quickly after addition to lake water.

### Spatial and temporal patterns of AgNPs

**Fig 2:**
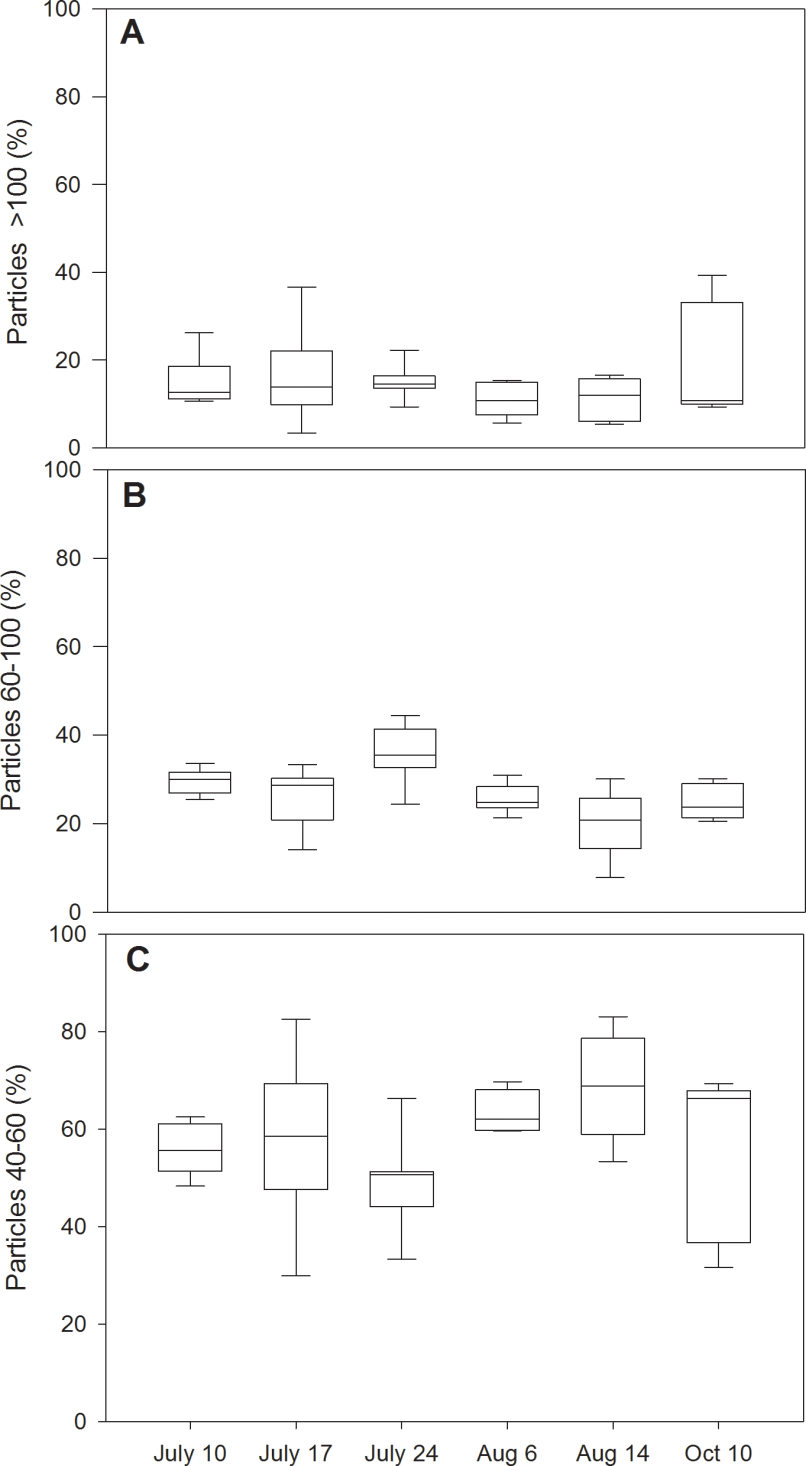
Box plots of Ag temporal variation among sampling dates. Ag size classes are presented as: (A) >100nm, (B) 40–100 nm, and (C) 40–60 nm sizes. Box plots are defined by 25^th^ and 75^th^ percentiles with a median line. Error bars represent 10^th^ to 90^th^ percentiles.

When comparing mean proportional occurrence across time and sampling location, sampling intervals varied slightly more than spatial location for 40–60 and 60–100 nm size classes while larger particles were more variable spatially (11–21%) than through time (10–17%) (Figs 1 and 2). These patterns captured by our synchrony analysis that assessed similarity among AgNP particle size classes across sites. We found statistically significant values for particles in the 40–60 nm (*S* = 0.24, *p* = 0.04) and 60–100 nm (*S* = 0.48, *p* = 0.004) sizes, but not for particles greater than 100 nm (*S* = 0.18, *p* = 0.07). These results indicate that the distribution of smaller sized particles was similar across vertical and horizontal locations during the study, despite thermal stratification that could have affected particle transport. Distributions also varied more across the surface of the lake than within the vertical profile (Figs 1 and 2). However, this difference was largely driven by increased variability near the point source where AgNP concentrations were highest.

The AgNP sizes in the lake were more variable by date than by spatial location (Figs 1 and 2). The higher temporal variability may be attributed to day to day variation in particle dispersal across the lake, as well as seasonal trends. For example, transport of particulate and dissolved material in lake environments is often related to turbulent mixing driven by weather conditions [45]. Within our study, the most variable dates for 40–60 nm and >100 nm sizes occurred on July 17 and October 10 (Fig 2). However, both days were characterized by higher proportions of particles >100 nm that were observed largely at the point source location, which may have contributed to this pattern.

Compared to previous fate assessments, AgNP size distributions in this study did not vary significantly with date. The continual daily addition of AgNPs during the experiment likely led to the consistent detection of all AgNP size classes during the study. Our previous findings in boreal lake mesocosms dosed with a single pulse addition of AgNPs indicated that larger aggregates and agglomerates (>60nm) were largely absent during the first 7 days of dosing [21]. The occurrence of larger particles that we observed through time may be attributed to the longer duration of this study or to the fact that real world conditions favor aggregate formation potentially by Ostwald ripening [13]. The large aggregates were most frequently detected at the point source, where approximately 30% of particles were greater than 100 nm at the highest event. This may also be due to the presence of larger aggregates in the original stock suspensions added to the lake. The overall conclusion from this study, however, is that the particles were remarkably evenly spread throughout the lake in both horizontal as well as vertical dimensions.

### Spatial and temporal trends of dissolved and total Ag

**Fig 3:**
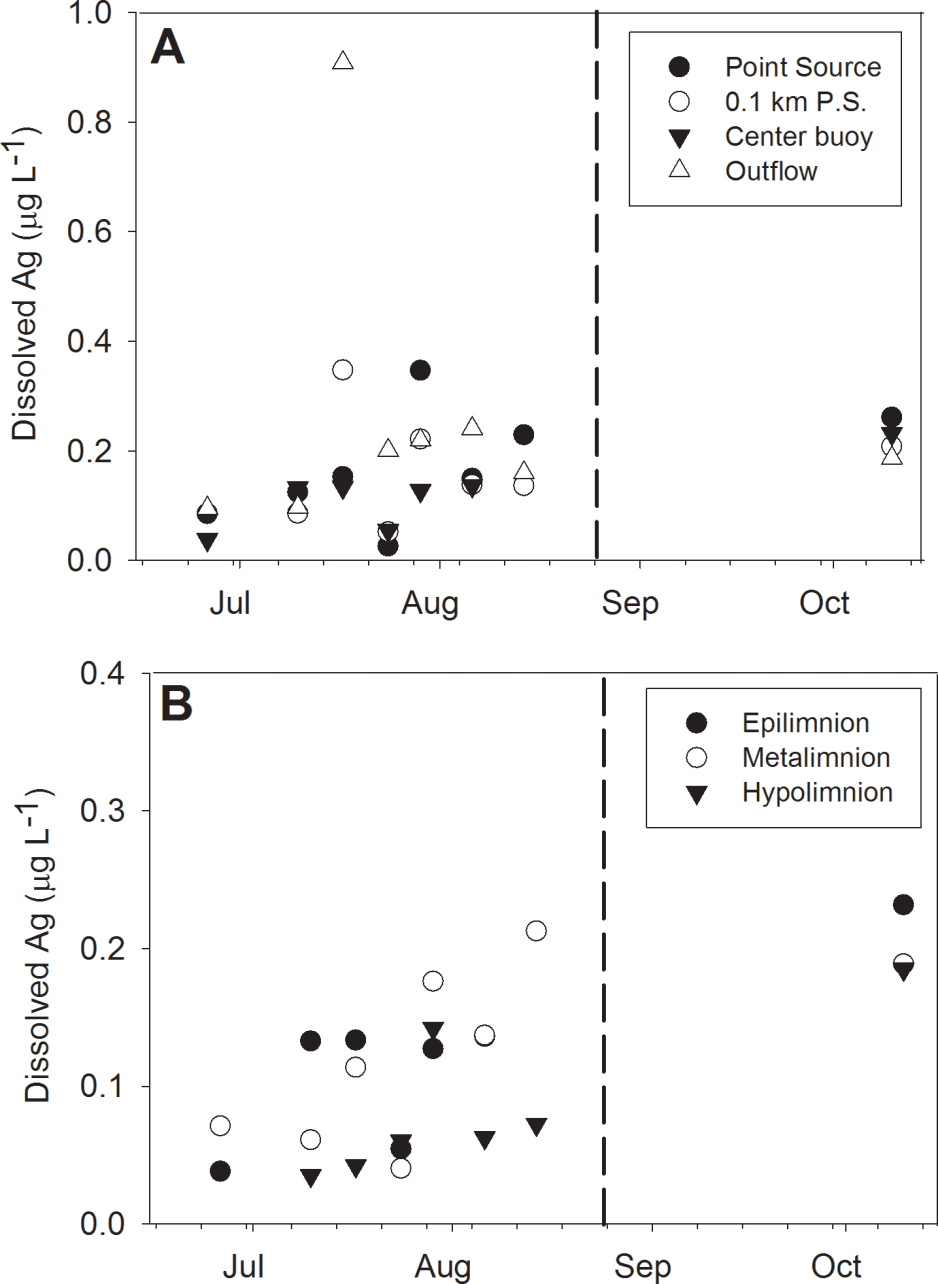
Spatial distribution of dissolved Ag concentrations (μg L^-1^). Dissolved Ag is presented as: A. horizontal and B. vertical lake gradients after AgNP addition in 2014. Dissolved Ag was determined during sp-ICP-MS as denoted by changes to background Ag content. Vertical dashed lines represent the fall turnover date in the lake.

Dissolved Ag (DAg) was typically detected at concentrations below 0.4 μg L^-1^ throughout the study (Fig 3). Our previous research with boreal lake mesocosms also showed that little DAg was present, which was attributed to a high degree of AgNP stability and the rapid formation of Ag-DOC complexes [21,22]. Given the elevated DOC concentrations and low ionic strength in Lake 222, our data of low DAg is consistent with these findings. After addition of AgNPs to the lake in mid-June, DAg increased slightly until a peak in August with a subsequent decline into October after lake stratification had deteriorated. Despite these slight changes, DAg was similar across sites as indicated by synchrony and permutation analyses (*S* = 0.44, *p* = 0.004). Similar to the AgNP sizes, DAg varied more across the surface water gradient than the vertical profile at the center of the lake (Fig 3A and 3B).

**Fig 4:**
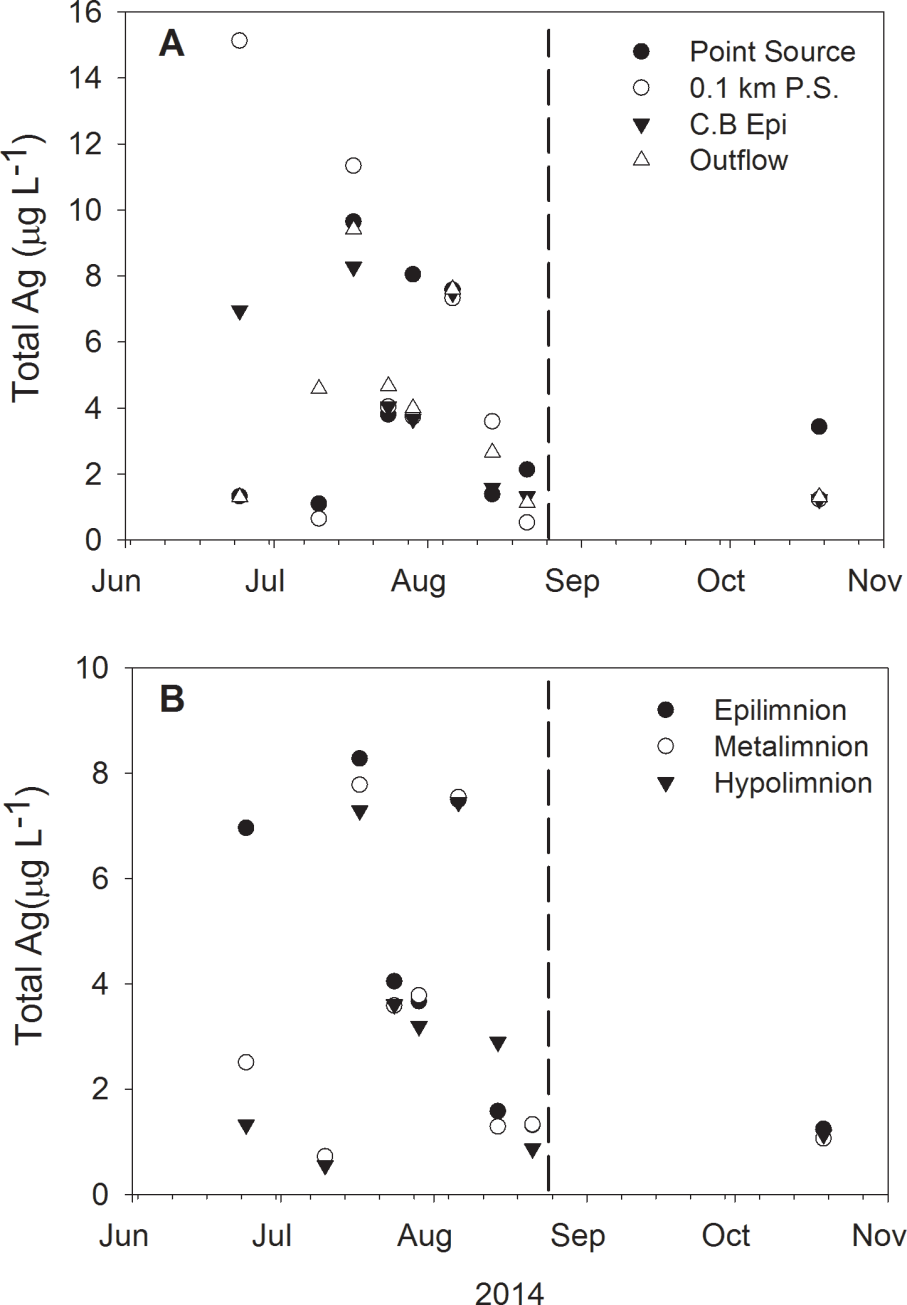
Spatial distribution of total Ag concentrations (μg L^-1^). Total Ag is presented as: (A) horizontal and (B) vertical lake gradients after AgNP addition in 2014. Vertical dashed lines represent the fall turnover date in the lake.

**Fig 5:**
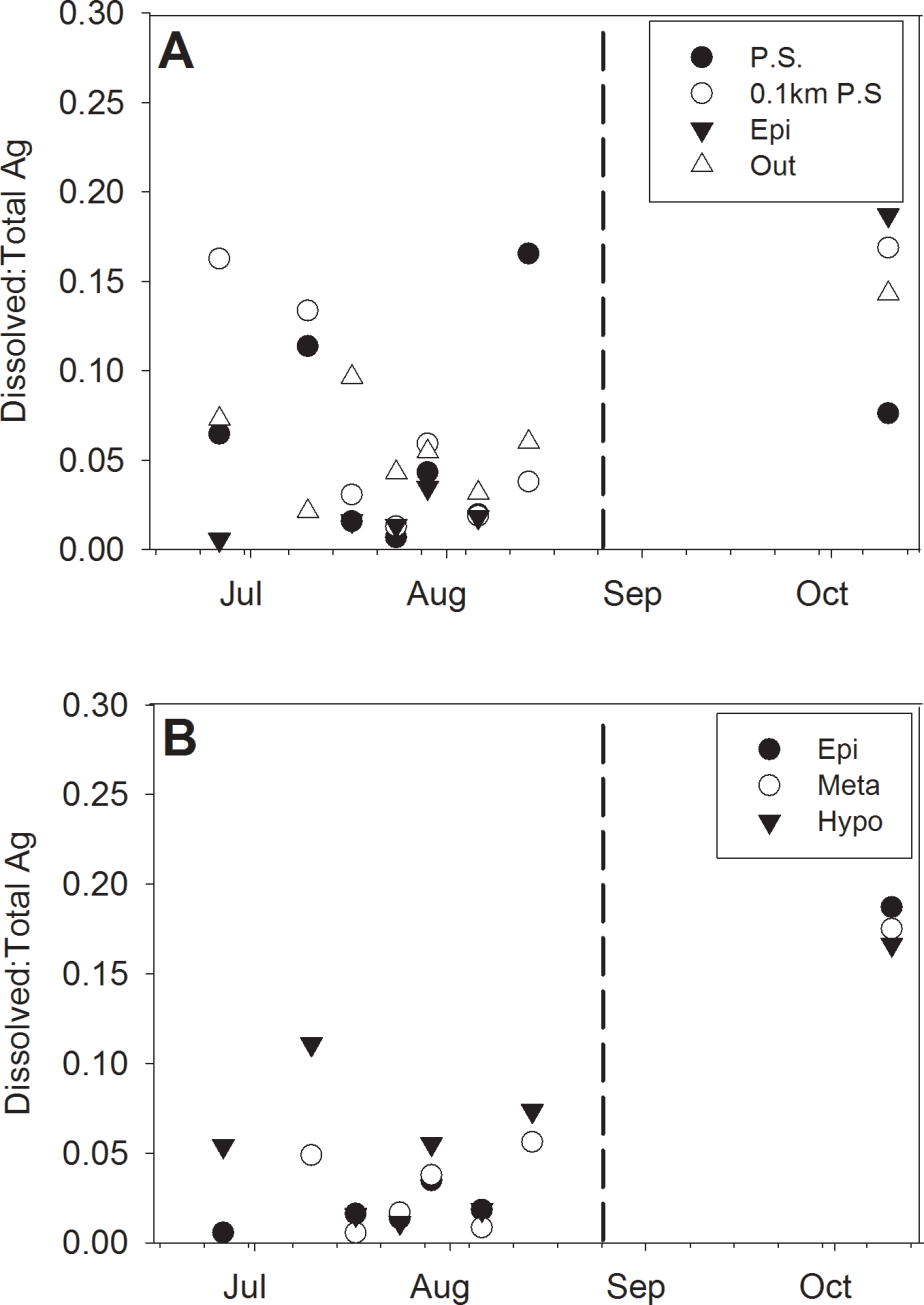
Dissolved to total Ag ratio detected across sampling locations and dates. Data are presented as: (A) surface linear gradient from the addition location, 0.1 km from P.S., center buoy, and the lake outflow, and (B) vertical gradient spanning the thermal lake profile from samples taken at the epilimnion, metalimnion, and hypolimnion. Vertical dashed lines represent the fall turnover date in the lake.

Total Ag was variable through time though generally sites were similar in a given day (Fig 4). Mean TAg was found to be 4.05 ± 3.28 μg L^-1^ during the study. When comparing TAg with DAg during the same sampling event, DAg was not found to be a major contributor to TAg. The concentration of DAg relative to TAg ranged from 0.006 to 0.18, with values generally occurring below 0.06 (Fig 5). Such low occurrence of DAg is consistent with findings in other mesocosms and microcosms [9,20]. Additionally, no discernible differences within spatial patterns were found among sites. The highest ratios occurred after fall turnover in mid-October, with mean values spanning 0.14 to 0.18.

### Physico-chemical drivers of particle distribution

**Fig 6:**
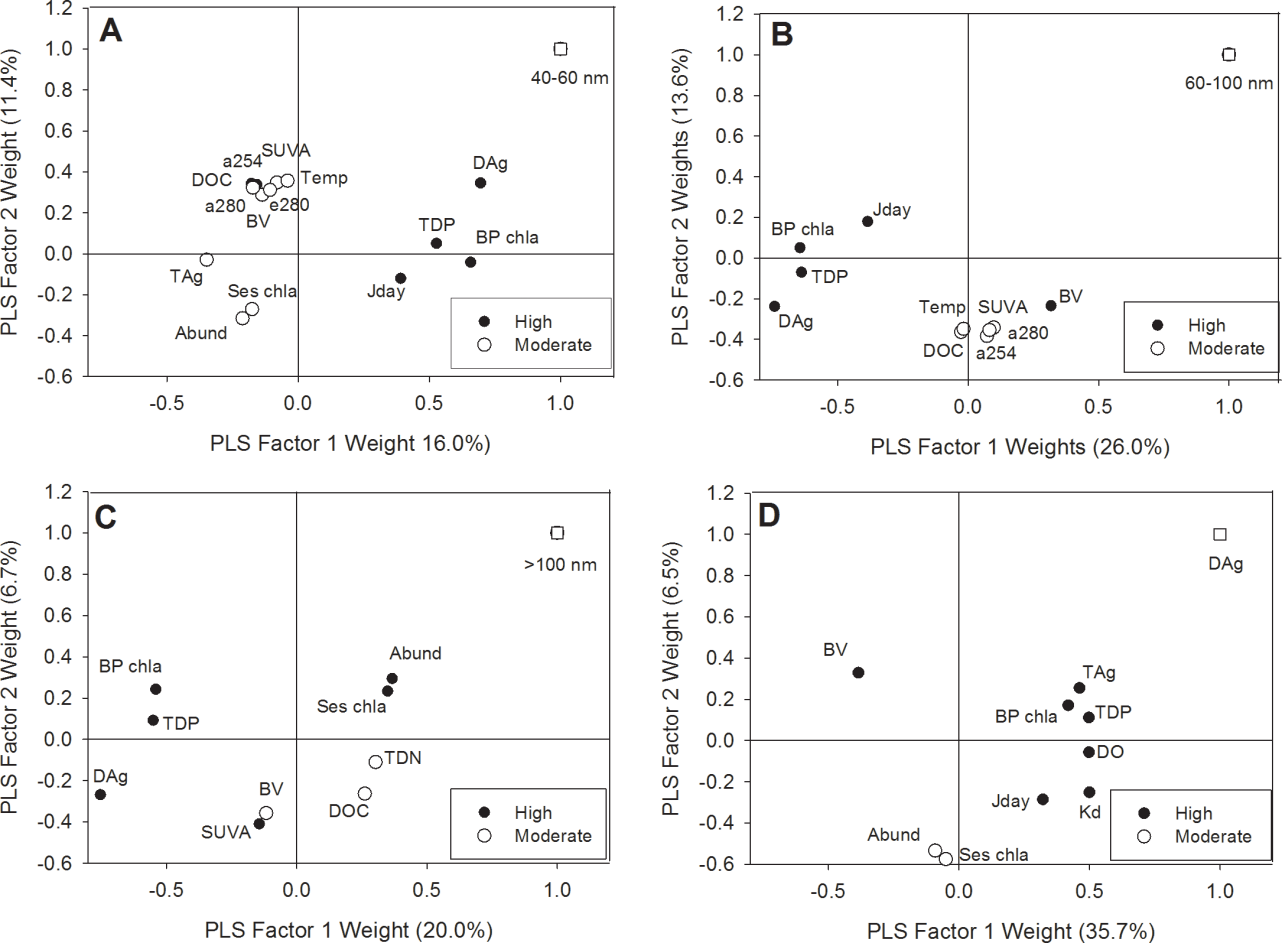
PLS regression plot of weights for Ag size classes with environmental factors. The figure shows the relationship between Ag size class (Y) with X-variables classified by their predictive influence using VIP scores. Data were defined as highly influential (black circles) and moderately influential (white circles) for X-variables with the predicted Y-variable designated as a white square. Subpanels represent size classes as: (A) 40–60 nm, (B) 60–100 nm, (C) >100 nm, and (D) Dissolved Ag. Criteria for influence strength were defined as highly influential (VIP>1) and moderately influential (VIP = 1 to 0.8) as established by Gudasz et al., 2012.

To determine environmental drivers controlling Ag particle distribution, we completed a PLS regression analysis for the different size classes observed in the study. Each size class (40–60 nm, 60–100 nm, >100nm, and DAg) was analyzed separately to assess the influence of environmental factors on each group. Several variables emerged as major predictors within the PLS models. For some models variation in Ag was weakly explained, suggesting parameters not measured in this study may play a significant role toward Ag form. Variables positively correlated with original nanoparticle form (40–60 nm) were generally negatively correlated with larger size classes. In the 40–60 nm model, 27% of the variation in Ag was accounted for by the included variables. While the analysis did not reveal strong relationships, the results provide some insight toward understanding AgNPs in an environmental setting across seasons. Increases in the 40–60 nm size distribution were positively correlated with DAg, TDP, BP Chl-a and Julian day, as indicated by highly influential VIP scores and position on the F1 axis (Fig 6A). The positive correlation with DAg is likely related to the persistence and stability of the AgNP form allowing for a higher percentage of dissolution to occur relative to larger aggregates with lower surface area to volume [46].

Other major contributors such as TDP and BP Chl-a were also found to be positively related to DAg, while inversely correlated with increasing 60–100 and >100 nm size classes of AgNPs. One explanation for this may relate to the role of phosphorus as a ligand in the environment. Phosphate has been shown to be a ligand capable of reducing toxicity associated with AgNPs and Ag^+^ [16,47,48]. Although with free phosphate concentrations at ELA in the ng L^-1^ range [49], it is unlikely that this ligand was a major contributor to stability. The positive correlation with BP Chl-A may be attributed to bacterial activity, altering phosphorus presence in the environment. One explanation may be that bacterioplankton often exude exopolymeric substances (EPS) that contain phosphate to meet their biological needs [50]. Production of EPS has been found to be upregulated in the presence of Ag [51]. As a form of natural organic material, these substances with DOC likely added to particle stability.

Our second PLS model assessed the 60–100 nm distribution of AgNP with environmental predictors. This model revealed DAg, TDP, and BP Chl-a were highly influential parameters aligning with the same factors that were most influential to the 40–60 nm model. However, unlike the 40–60 nm model, these factors were inversely proportional to the 60–100 nm size (Fig 6B). In comparison, the 60–100 nm model was a better fit with 43% of the variation explained by the first two factors. Other moderate predictors included Julian day (negative) and TAg (positive). The >100 nm PLS model illustrated these factors were also good predictors of this size class (Fig 6C) with increasing particles inversely proportional to DAg, TDP, and BP chl-a. These data suggest phosphorus ligands originating from EPS or other sources seasonally may affect aggregation in a lake environment. Further research is needed to clarify this phenomenon. Environmental factors such as seston chl-a, DOC, and TDN were also positively related but less influential to the PLS model, with the selected variables accounting for 26.7% of the variability seen in larger aggregate distribution.

Major predictors for DAg included dissolved oxygen (DO), TDP, and BP chl-a. All of the highly influential predictors were positively correlated with DAg with the model explaining 42% of the variability (Fig 6D). Similarities between AgNP form and DAg models are likely related to dissolution kinetics of AgNPs affecting DAg occurrence. Increased surface area to volume ratios of the AgNP form often generate higher rates of dissolution than larger aggregate counterparts [46]. Remarkably, DO was highly influential to DAg but not for other AgNP forms, that were detected more frequently in the lake. DO and temperature varied with depth and season, as seen in the lake profiles (S2 Fig). In the presence of DO AgNP dissolution can increase via oxidative processes as seen in declining nanoparticle diameters [52]. AgNP induced dissolution via oxidative processes often occurs slowly with total dissolution in laboratory settings ranging 6 to 125 days for citrate capped AgNPs [14]. Despite differences in capping agent and environmental context, DO concentrations remain strong drivers of DAg.

## Conclusions

This study showed that AgNPs were rapidly dispersed in a lake that was dosed at a point source along the lakeshore. Particles remained predominantly in the original size range of 40–60 nm over several months, with intermediate (60–100 nm) and larger (>100 nm) particles occurring less frequently. The temperature gradient at the thermocline did not appear to be a significant barrier to the dispersal of the AgNPs into the hypolimnion. The consistent AgNP size distributions and the low DAg concentrations detected across sampling locations and seasons indicate that dissolved organic matter and other ligands contribute to the stability of the nanoparticles. This may have implications for the toxicity to aquatic organisms, as toxicity is related to concentrations of Ag^+^ released by particle dissolution [53]. The environmental significance of these data are that AgNPs released into some aquatic environments may show a high degree of mobility and persistence. We chose to add the AgNPs at a point source along the shore to mimic the discharges that could occur from treated municipal wastewater. The low ionic strength and the high DOC levels in the study lake contributed to the stability of the AgNPs and the capacity of these particles to move rapidly throughout the lake.

Abiotic factors that have been shown to influence aggregation and dissolution kinetics in laboratory assessments, including pH [11,14], light [17,46], and DOC [13,14] were not strong predictors of changes in particle size. However, these parameters did not vary appreciably across sites or temporally in the current study. For instance, DOC concentrations varied from 9–16 mg L^-1^ across the sampling period, and other parameters were even less variable. Instead, biological parameters such as BP Chl-a may better explain size distributions due to significant changes through seasons. The PLS regression models show that seasonal variation among such biological factors may influence AgNP transformation, in turn affecting removal from the water column.

## Supporting information

S1 Fig Example size distribution histogram of particle diameters plotted with events detected from a single sample. Data were binned by 2 nm increments and later classified as 40–60, 60–100, or >100 nm for statistical analysis.
(PDF)

S2 Fig Lake 222 Temperature and dissolved oxygen profiles. **A.** Temperature (°C) and **B.** dissolved oxygen (mg L^-1^) profiles of Lake 222 during the 2014 sampling.
(PDF)
