# Supplementary material for: Spatial and temporal trends in the fate of silver nanoparticles in a whole-lake addition study
Source: PLoS One. 2018 Aug 15;13(8):e0201412. doi: 10.1371/journal.pone.0201412 (PMC6093604; doi:10.1371/journal.pone.0201412)
Supplement: S1 Fig — Data were binned by 2 nm increments and later classified as 40–60, 60–100, or >100 nm for statistical analysis. (PDF) [file pone.0201412.s001.pdf]

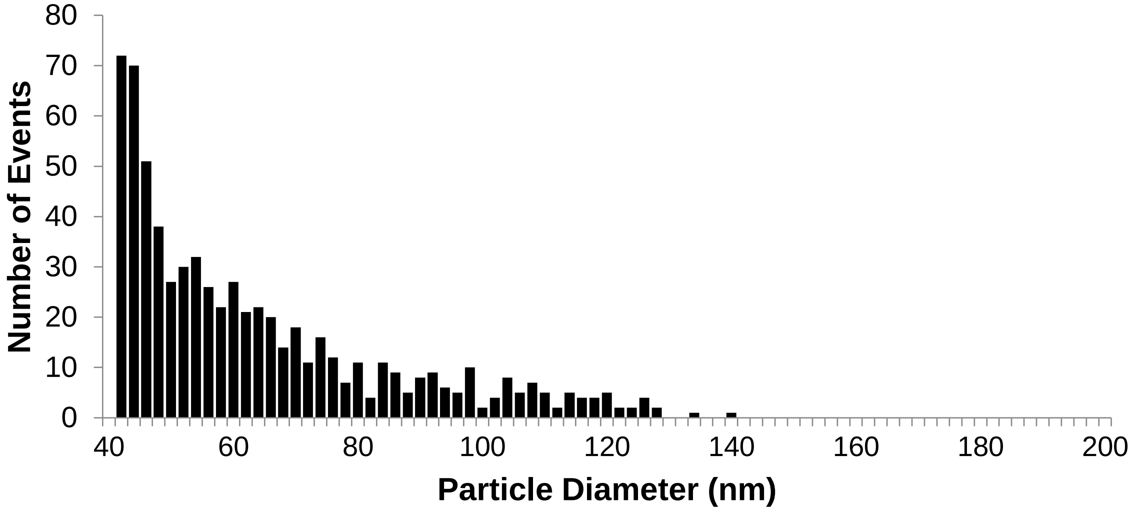

S1 Fig. **Example size distribution histogram of particle diameters plotted with events detected from a single sample.** Data were binned by 2 nm increments and later classified as 40-60, 60-100, or >100 nm for statistical analysis.
