# Supplementary material for: Spatial and temporal trends in the fate of silver nanoparticles in a whole-lake addition study
Source: PLoS One. 2018 Aug 15;13(8):e0201412. doi: 10.1371/journal.pone.0201412 (PMC6093604; doi:10.1371/journal.pone.0201412)
Supplement: S2 Fig — A. Temperature (°C) and B. dissolved oxygen (mg L-1) profiles of Lake 222 during the 2014 sampling. (PDF) [file pone.0201412.s002.pdf]

A

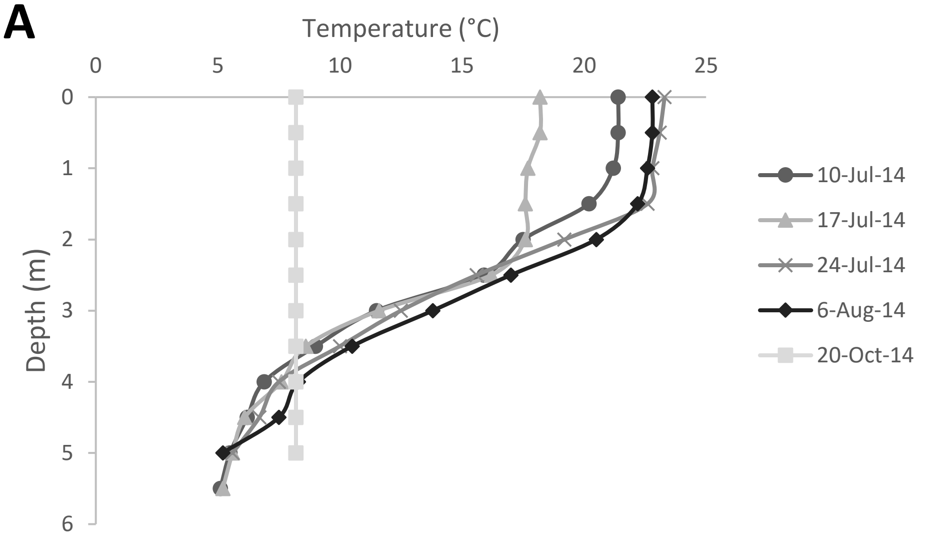

B

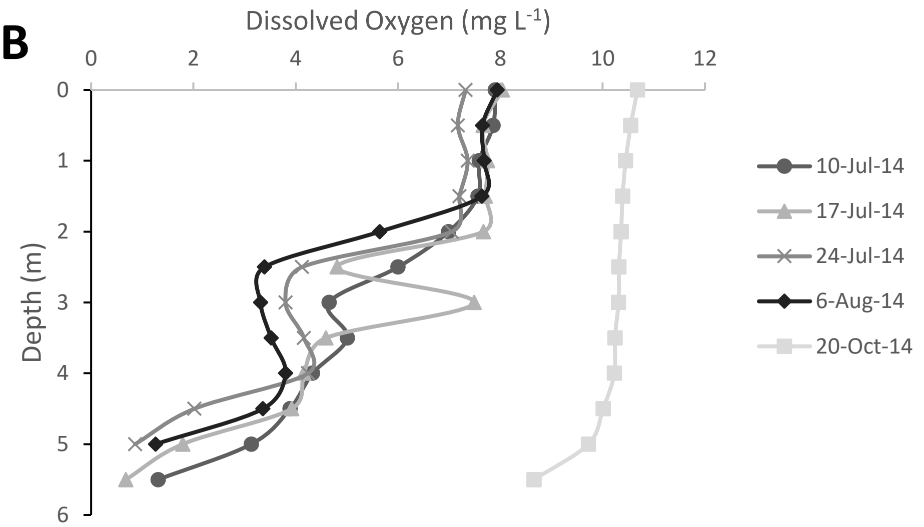

S2 Fig. **Lake 222 Temperature and dissolved oxygen profiles. A.** Temperature (°C) and **B.** dissolved oxygen (mg L^{-1}) profiles of Lake 222 during the 2014 sampling.
